# Loss-of-function G6PD variant moderated high-fat diet-induced obesity, adipocyte hypertrophy, and fatty liver in male rats

**DOI:** 10.1016/j.jbc.2024.107460

**Published:** 2024-06-12

**Authors:** Shun Matsumura, Christina Signoretti, Samuel Fatehi, Bat Ider Tumenbayar, Catherine D’Addario, Erik Nimmer, Colin Thomas, Trisha Viswanathan, Alexandra Wolf, Victor Garcia, Petra Rocic, Yongho Bae, SM Shafiqul Alam, Sachin A. Gupte

**Affiliations:** 1 Department of Pharmacology, New York Medical College, Valhalla, New York, USA; 2 Department of Pharmacology and Toxicology, Jacobs School of Medicine and Biomedical Sciences, University at Buffalo, State University of New York, Buffalo, New York, USA; 3 Department of Biomedical Engineering, School of Engineering and Applied Sciences, University at Buffalo, State University of New York, Buffalo, New York, USA; 4 Department of Physiology & Pharmacology, SHSU College of Osteopathic Medicine, Conroe, Texas, USA; 5 Department of Pathology and Anatomical Sciences, Jacobs School of Medicine and Biomedical Sciences, University at Buffalo, State University of New York, Buffalo, New York, USA; 6 Department of Pathology, Microbiology, and Immunology (PMI), New York Medical College, Valhalla, New York, USA

**Keywords:** metabolic reprogramming, inflammation, inter-organ communication, cytokines, chemokines, vascular biology, fat tissue, liver

## Abstract

Obesity is a major risk factor for liver and cardiovascular diseases. However, obesity-driven mechanisms that contribute to the pathogenesis of multiple organ diseases are still obscure and treatment is inadequate. We hypothesized that increased , glucose-6-phosphate dehydrogenase (G6PD), the key rate-limiting enzyme in the pentose shunt, is critical in evoking metabolic reprogramming in multiple organs and is a significant contributor to the pathogenesis of liver and cardiovascular diseases. G6PD is induced by a carbohydrate-rich diet and insulin. Long-term (8 months) high-fat diet (HFD) feeding increased body weight and elicited metabolic reprogramming in visceral fat, liver, and aorta, of the wild-type rats. In addition, HFD increased inflammatory chemokines in visceral fat. Interestingly, CRISPR-edited loss-of-function Mediterranean G6PD variant (G6PD^S188F^) rats, which mimic human polymorphism, moderated HFD-induced weight gain and metabolic reprogramming in visceral fat, liver, and aorta. The G6PD^S188F^ variant prevented HFD-induced CCL7 and adipocyte hypertrophy. Furthermore, the G6PD^S188F^ variant increased *Magel2* – a gene encoding circadian clock-related protein that suppresses obesity associated with Prader-Willi syndrome – and reduced HFD-induced non-alcoholic fatty liver. Additionally, the G6PD^S188F^ variant reduced aging-induced aortic stiffening. Our findings suggest G6PD is a regulator of HFD-induced obesity, adipocyte hypertrophy, and fatty liver.

Obesity is an emerging global pandemic. CDC estimates suggest roughly half of US adults are likely to become obese by 2030. Overweight and obesity are caused by a variety of factors including high-fat diet (HFD)/calorific diet, sedentary lifestyle, stress, medical conditions, and genetics. It is a significant risk factor for vascular, heart, liver, and kidney diseases, often with inadequate treatment. It is estimated that obesity increases the risk of cardiovascular diseases by 28%, kidney diseases by 24 to 33%, and liver diseases by 65 to 80%, compared to the non-obese population (1, 2, 3). Therefore, it is of paramount importance to determine mechanisms (or factors) contributing to the pathogenesis of obesity-associated multi-organ diseases, so that new therapies can be developed to mitigate obesity-connected pathologies.

Recent studies suggest that disturbances in interorgan communication, vital to maintaining homeostatic balance in a physiological state, contribute to an array of diseases (4). In this context, perivascular visceral fat augments coronary and mesenteric artery contraction in obese pigs (5), and obesity-connected nonalcoholic fatty liver (NAFL) has been associated with arterial stiffness and endothelial/microvascular dysfunction in humans (6, 7). Arterial stiffness is a major risk factor for hypertension, heart failure, and organ damage (8). It is affected by aging, metabolic diseases (including diabetes, obesity, homocysteinemia, and hypercholesterolemia), and connective tissue diseases, including the Marfan Syndrome (8, 9, 10). In addition, it appears that there is a dichotomy between the mechanisms of arterial stiffness observed in young *versus* old mice (11). To date, although extensive research has been conducted to identify the risk factor(s) and elucidate the mechanism(s) responsible for the pathogenesis of arterial stiffness, our knowledge regarding the factors released from perivascular visceral adipose tissue (PVAT) and/or NAFL for interorgan communication and pathophysiological mechanisms responsible for arterial stiffening associated with obesity and aging remains incomplete. Therefore, our first objective was to determine whether adipose tissue inflammation or NAFL elicits arterial stiffness in HFD-induced obese rats.

Metabolic reprogramming is evolving as a central player in the pathology of liver, heart, and vascular diseases (12, 13). While increased aerobic glycolysis is implicated in the pathogenesis of non-vascular and vascular diseases, glucose-6-phosphate dehydrogenase (G6PD)—the key rate-limiting enzyme in the pentose shunt—expression and activity are increased in liver and adipose tissue by carbohydrate-rich diet, hyperinsulinemia, oxidants, glutathione redox, and NADPH (14, 15, 16). Besides, G6PD has been associated with HFD-induced adipocyte inflammation (17). In contrast, G6PD deficiency in mice has been shown to decrease weight gain and hyperinsulinemia and to modestly suppress glucose flux into nonoxidative pathways in myocardium associated with obesogenic diet (18). Interestingly, G6PD deficiency in mice reduces angiotensin II-induced hypertension and atherosclerosis (19, 20), and a loss-of-function Mediterranean G6PD (G6PD^S188F^) variant in rat reduces hypertension and large artery stiffness induced by feeding of HFD/obesogenic diet for 4 months (21). But several questions remain unanswered: does G6PD^S188F^ variant moderate (1) metabolic reprogramming in various organs; (2) adipose tissue inflammation; (3) NAFL disease; (4) arterial stiffness/vasculopathies; and (5) heart and kidney failure, in rats fed with HFD/obesogenic diet for long-term (8 months). More importantly, does G6PD^S188F^ variant restore inter-organ communication homeostasis in HFD/obesogenic diet-fed rats? Our results suggest that the G6PD^S188F^ variant reduced HFD-induced weight gain/obesity, adipocyte hypertrophy, and NAFL. Furthermore, our results suggest, for the first time, that HFD-induced adipose tissue inflammatory chemokines (CCL5), at least partly, contributed to arterial stiffening.

## Results

### G6PD^S188F^ variant reduces HFD-induced weight gain and visceral adipose cell hypertrophy

**Figure 1:**
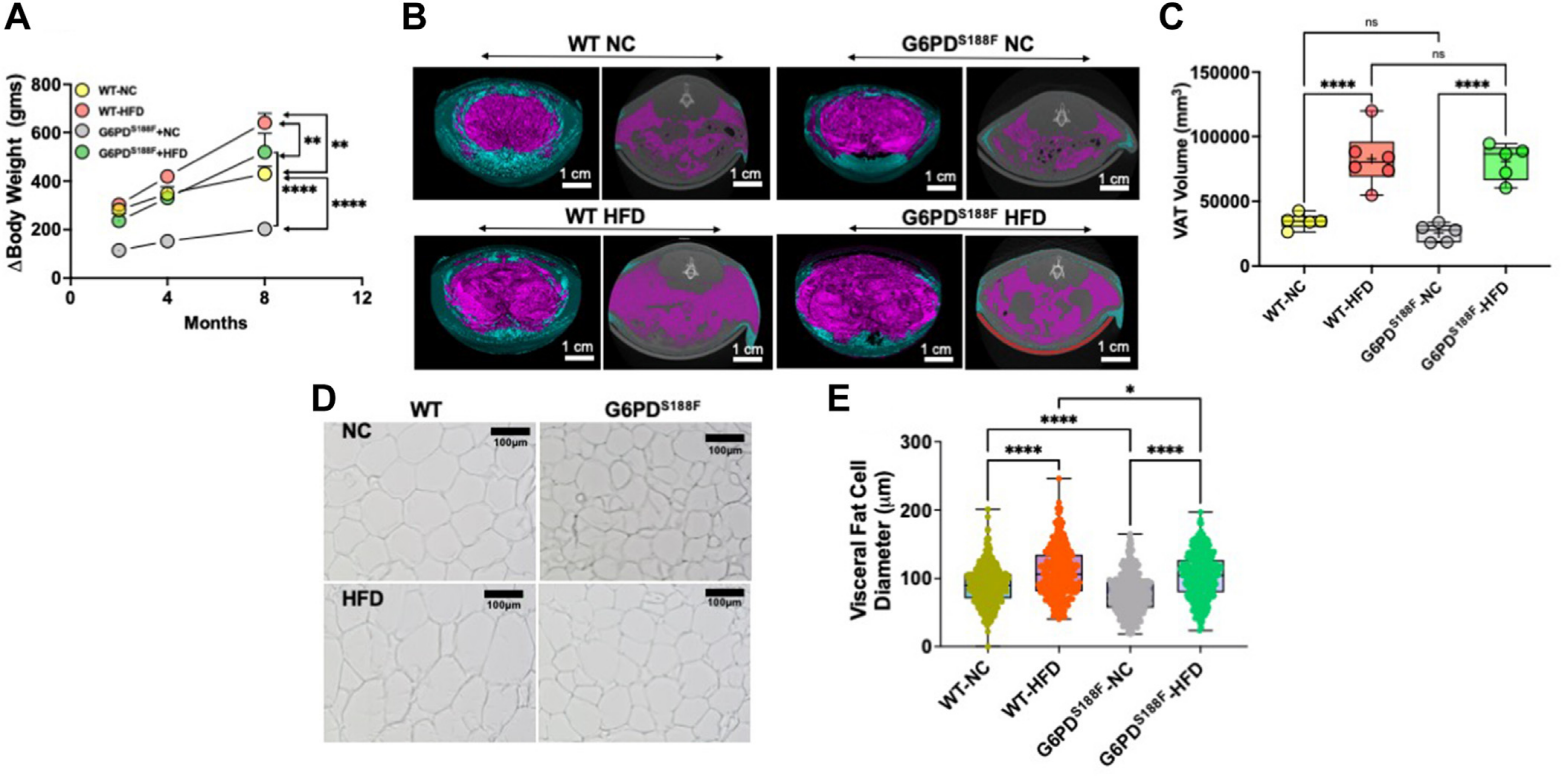
**Effect of long-term high-fat diet feeding on body weight and visceral adipose tissue of wild-type and G6PD**^**S188F**^**rats.***A*, difference in body weight of wild-type (WT) and G6PD^S188F^ rats before and after feeding high-fat diet (HFD) show body weight increased in HFD fed (WT-HFD and G6PD^S188F^-HFD) groups compared to their respective NC diet fed (WT-NC and G6PD^S188F^-NC) groups. However, G6PD^S188F^-HFD rats gained less weight than WT-HFD rats. *B* and *C*, two representative micro-CT scans of wild-type and G6PD^S188F^ rats on NC and HFD showed visceral (*pink*) and subcutaneous (*blue*) adipose tissue, and summary results showing visceral adipose tissue volume increased in both the genotypes on HFD. *D* and *E*, representative images and summary results of visceral adipose cell sizes demonstrate cell size is increased in WT-HFD but not G6PD^S188F^-HFD rats as compared with the respective controls (NC). N = 5 in *panel A*–*D* and individual cells from five different samples. Two-way ANOVA with *post hoc* Tukey’s multiple comparison tests was used to compare multiple groups. ∗*p* < 0.05; ∗∗*p* < 0.01; ∗∗∗∗*p* < 0.001.

*G6pd* is an X-linked gene and because males are affected by loss-of-function polymorphism of *G6pd* gene, in this study, we used male G6PD^S188F^ variant rats and their age-matched wild-type littermates. Our results revealed that long-term (for 8 months) HFD feeding increased body weight in both genotypes. As expected, HFD-fed wild-type rats gained more weight than NC diet-fed rats. Interestingly, HFD-fed G6PD^S188F^ variant rats gained significantly less body weight than HFD-fed wild-type rats (Fig. 1*A*). Micro-CT results showed that the visceral adipose tissue (VAT) and subcutaneous adipose tissue (SAT) volume increased, respectively, by 2.0-fold and 3.8-fold in wild-type rats and by 1.0-fold and 1.1-fold in G6PD^S188F^ rats in age-dependent manner (from 4 to 8 months) and on NC diet. Although a comparison of lipid volume using micro-CT showed HFD increased VAT volume in both genotypes (Fig. 1, *B* and *C*), adipocyte diameter was significantly smaller in VAT of G6PD^S188F^ than wild-type rats fed with NC and HFD (Fig. 1, *D* and *E*). However, SAT volume was less (*p* < 0.05) in HFD-fed G6PD^S188F^ rats (5507 ± 855 mm^3^; N = 5) than in the wild-type littermates (11,011 ± 2468 mm^3^; N = 5). Furthermore, HFD induced dyslipidemia in both genotypes but total cholesterol levels in serum were significantly less in G6PD^S188F^ than in wild-type rats fed with NC and HFD (Table 1). Even though total cholesterol levels were reduced, we found approximately 50% more LDL and 41% less HDL in serum from G6PD^S188F^ than wild-type rats fed with HFD (Table 1). Also, we found 35% (not significant) less HDL in the serum of G6PD^S188F^ than in wild-type rats on NC (Table 1).

**Table 1:** Blood glucose, liver enzymes, and lipid profile, in wild-type and G6PD^S188F^ rats fed with a high-fat diet for 8 months

Blood content non-fasting	WT+NC	WT+HFD	G6PD^S188F^+NC	G6PD^S188F^+HFD
Glucose (mg/dl)	367.2 ± 57.3	363.2 ± 45.4	332.4 ± 13.8	291.6 ± 13.1
ALT (U/L)	95.8 ± 18.0	73.5 ± 6.5	81.0 ± 9.0	65.2 ± 8.9
AST (U/L)	301.4 ± 28.0	295.2 ± 33.8	354.2 ± 90.2	289.6 ± 66.1
T-Cholesterol (mg/dl)	85.6 ± 16.2	111.7 ± 11.5	63.8 ± 6.0	86.4 ± 5.3^a^
Triglycerides (mg/dl)	184.2 ± 38.5	160.5 ± 20.0	140.0 ± 9.3	126.0 ± 5.9
HDL (mg/dl)	50.6 ± 10.0	64.8 ± 9.6	32.8 ± 5.6	38.4 ± 4.9^a^
nHDLc (mg/dl)	34.8 ± 6.2	46.8 ± 3.5^b^	31.0 ± 2.6	48.2 ± 1.5^c^
LDL (mg/dl)	3.4 ± 2.1	14.7 ± 2.3^d^	5.0 ± 2.2	23.0 ± 0.6^c^^a^
VLDL (mg/dl)	37.0 ± 7.7	32.0 ± 3.9	27.8 ± 1.9	25.2 ± 1.2

Mean ± SEM.

a *p* < 0.05 *versus* WT+HFD.

b *p* < 0.05 *versus* WT+NC or G6PD^S188F^.

c *p* < 0.0005 *versus* WT+NC or G6PD^S188F^.

d *p* < 0.005 *versus* WT+NC or G6PD^S188F^.

### HFD increases G6PD activity and alters metabolism in VAT

**Figure 2:**
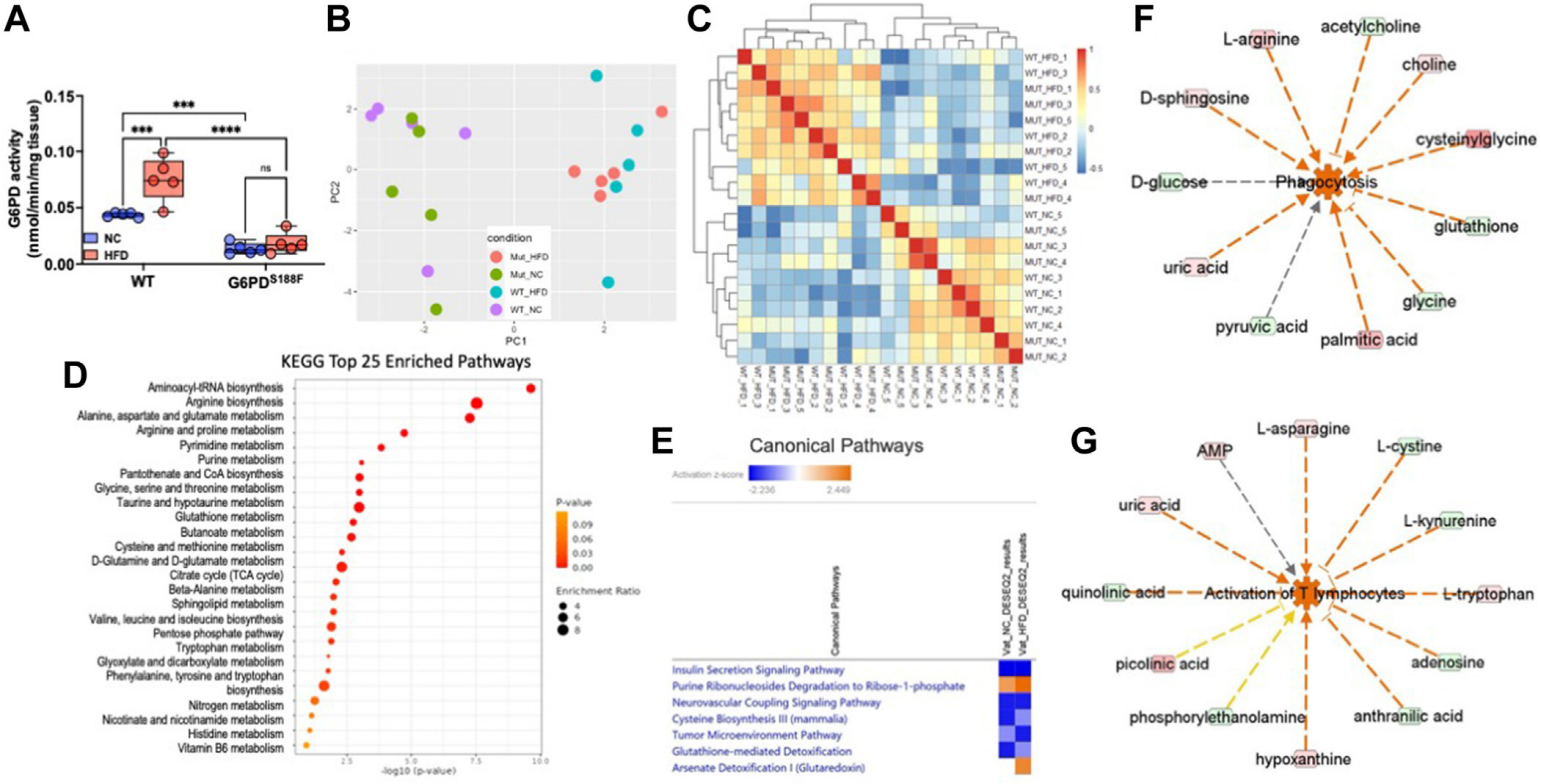
**Effect of long-term high-fat diet feeding on G6PD activity and metabolomic reprogramming, and inflammatory chemokines synthesis, in visceral adipose tissue of wild-type and G6PD**^**S188F**^**rats.***A*, G6PD activity increased in visceral adipose tissue (VAT) of wild-type (WT) rats but not G6PD^S188F^ rats fed with HFD. *B* and *C*, PCA plot and sample correlation heat map demonstrating differential metabolism in VAT of WT and G6PD^S188F^ fed with normal chow (NC) and high fat diet (HFD). *D*, KEGG enrichment pathway analysis identified the top 25 pathways in response to G6PD mutation. *E*, IPA core analysis predicted seven canonical pathways are significantly and differentially (Absolute z-score ≥ 2 and log10(*p*-value) ≥ 1.3) changed in response to G6PD mutation. *F* and *G*, IPA disease and function network analysis of metabolomic results predicted that a number of inflammatory response functions are activated in response to G6PD mutation. The majority of inflammatory responses including phagocytosis, activation of T lymphocytes, and immune response of cells changed more in HFD when compared to normal chow. Prediction legends show various symbols and arrows for interpretation of the IPA analysis. N = 5 in each group. Two-way ANOVA with *post hoc* Tukey’s multiple comparison test were used to compare multiple groups. ∗*p* < 0.05; ∗∗*p* < 0.01; ∗∗∗*p* < 0.005; and ∗∗∗∗*p* < 0.001.

Since hormones and diet regulate G6PD activity, and increased G6PD activity regulates fatty acid metabolism (16), we determined G6PD activity and metabolism in VAT. While VAT and SAT volume increased in wild-type rats on the NC diet in an age-dependent manner, we did not find significant changes in the G6PD activity and metabolism (data not shown). However, HFD for 8 months increased G6PD activity in VAT of wild-type rats but not in G6PD^S188F^ rats (Fig. 2*A*). Next, we performed unbiased metabolomic analysis on VAT samples as described previously (22, 23, 24). Interestingly, principal component analysis plot and sample correlation heat map of unbiased metabolomic revealed that metabolic phenotype was altered in VAT of HFD-fed wild-type and G6PD^S188F^ rats (Fig. 2, *B* and *C*). KEGG enrichment pathway analysis suggested glutathione homeostasis; glycolysis; TCA cycle; PPP; glutamyl pathway; polyamine pathway; sulfur metabolism; indole and tryptophan metabolism; and fatty acid oxidation were altered by HFD feeding in VAT (Fig. 2*D*), and were more altered in VAT of wild-type rats than G6PD^S188F^ rats (Fig. S1*A*). In addition, SMPDB enrichment pathway analysis identified alterations in similar pathways (Fig. S1*B*). Further, IPA canonical pathway function analysis predicted seven canonical pathways were significantly and differentially changed ([threshold: absolute z-score ≥2 and -log10 (*p*-value) ≥1.3]) in response to G6PD mutation (Fig. 2*E*). In IPA core upstream analysis, 40 upstream molecules were predicted to be significantly and differentially modified [threshold: absolute z-score ≥2 and -log10 (*p*-value) ≥1.3] in response to G6PD mutation (Fig. S1*C*).

### HFD increases inflammatory chemokines in VAT

**Figure 3:**
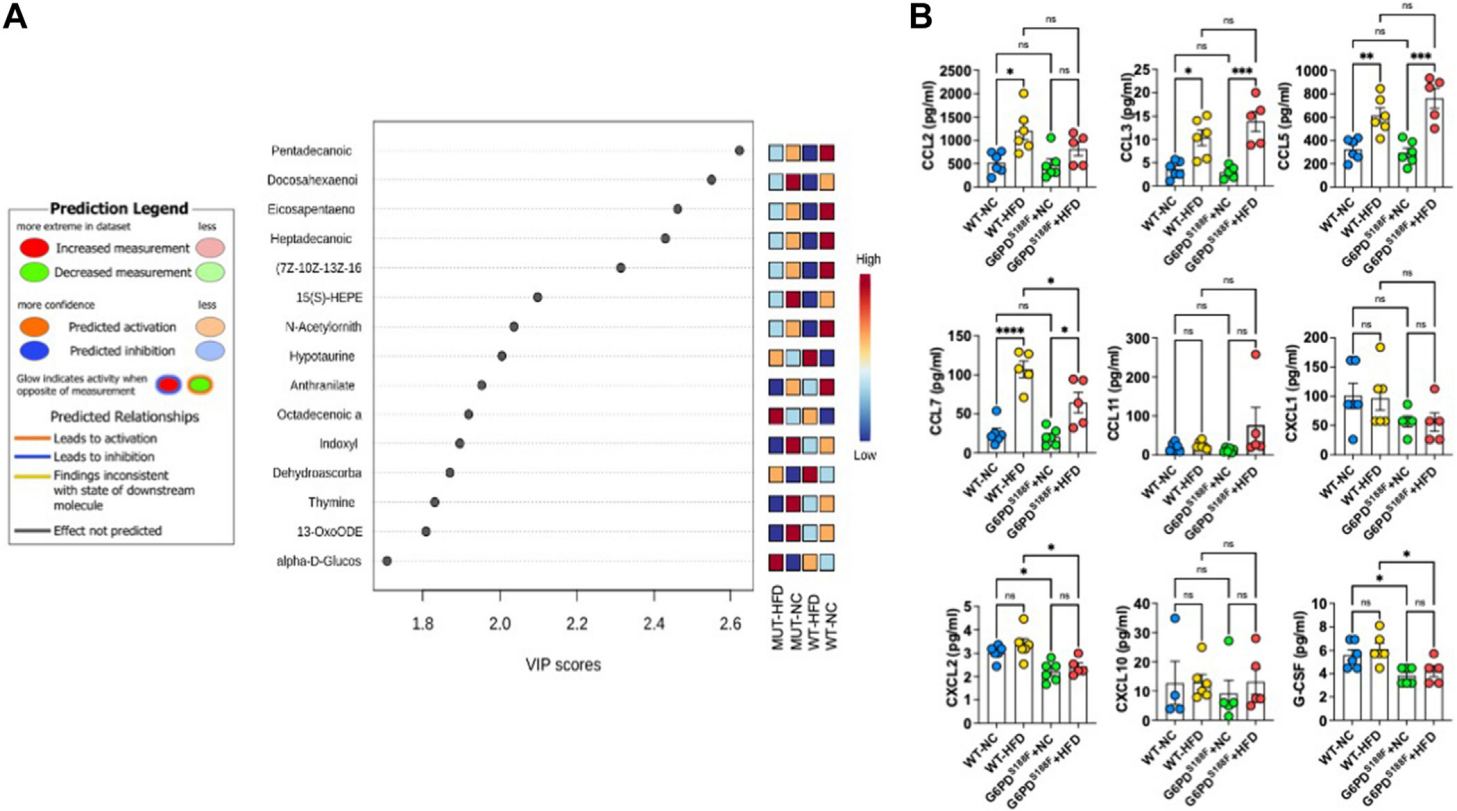
**Effect of long-term high-fat diet feeding on inflammatory chemokines synthesis in visceral adipose tissue of wild-type and G6PD**^**S188F**^**rats.***A*, VIP score plot shows metabolites including oxylipins differed in HFD from NC group. *B*, summary results of multiplex analysis demonstrate chemokines are increased in visceral adipose tissue of WT and G6PD^S188F^ rats. N = 5 in each group. Two-way ANOVA with *post hoc* Tukey’s multiple comparison test were used to compare multiple groups. ∗*p* < 0.05; ∗∗*p* < 0.01; ∗∗∗*p* < 0.005; and ∗∗∗∗*p* < 0.001.

IPA network analysis predicted numerous diseases and functions changed [threshold: absolute z-score ≥2 and -log10 (*p*-value) ≥1.3] in response to G6PD mutation (Fig. S2*A*). The majority of inflammatory responses, including phagocytosis, activation of T lymphocytes, and immune response of cells, changed more in HFD when compared to NC (Fig. 2, *F* and *G*). Furthermore, IPA network analysis identified two networks in the NC diet related to inflammatory disease, inflammatory response, and organismal injury and abnormalities, and one network in the HFD group related to immunological disease, inflammatory disease, and inflammatory response (Fig. S2*B*). Further, the VIP score plot showed oxylipins, particularly eicosapentaenoic acid and 15(S)-HEPE, significantly decreased in VAT by HFD feeding (Figs. 3*A* and S1*D*). 15(S)-HEPE suppresses leukotriene B4-induced chemotaxis of polymorphonuclear leukocytes (25, 26). Previous studies have shown that overexpression of G6PD evokes an inflammatory response in adipose tissue (27). Therefore, we measured cytokines in VAT of wild-type and G6PD^S188F^ variant rats fed with NC and HFD by Multiplex assay. We found that HFD feeding increased C-C motif chemokines (CCL3, CCL5, and CCL7) in VAT both genotypes (Fig. 3*B*). However, HFD-induced CCL2 increase was prevented in VAT of G6PD^S188F^ rats, and HFD-induced CCL7 increase was attenuated in VAT of G6PD^S188F^ rats as compared to their wild-type littermates (Fig. 3*B*). Furthermore, we found that CXCL2 and G-CSF levels were lower in VAT collected from G6PD^S188F^ rats than wild-type rats fed with NC and HFD (Fig. 3*B*).

### HFD increases G6PD activity and alters metabolism in the liver

**Figure 4:**
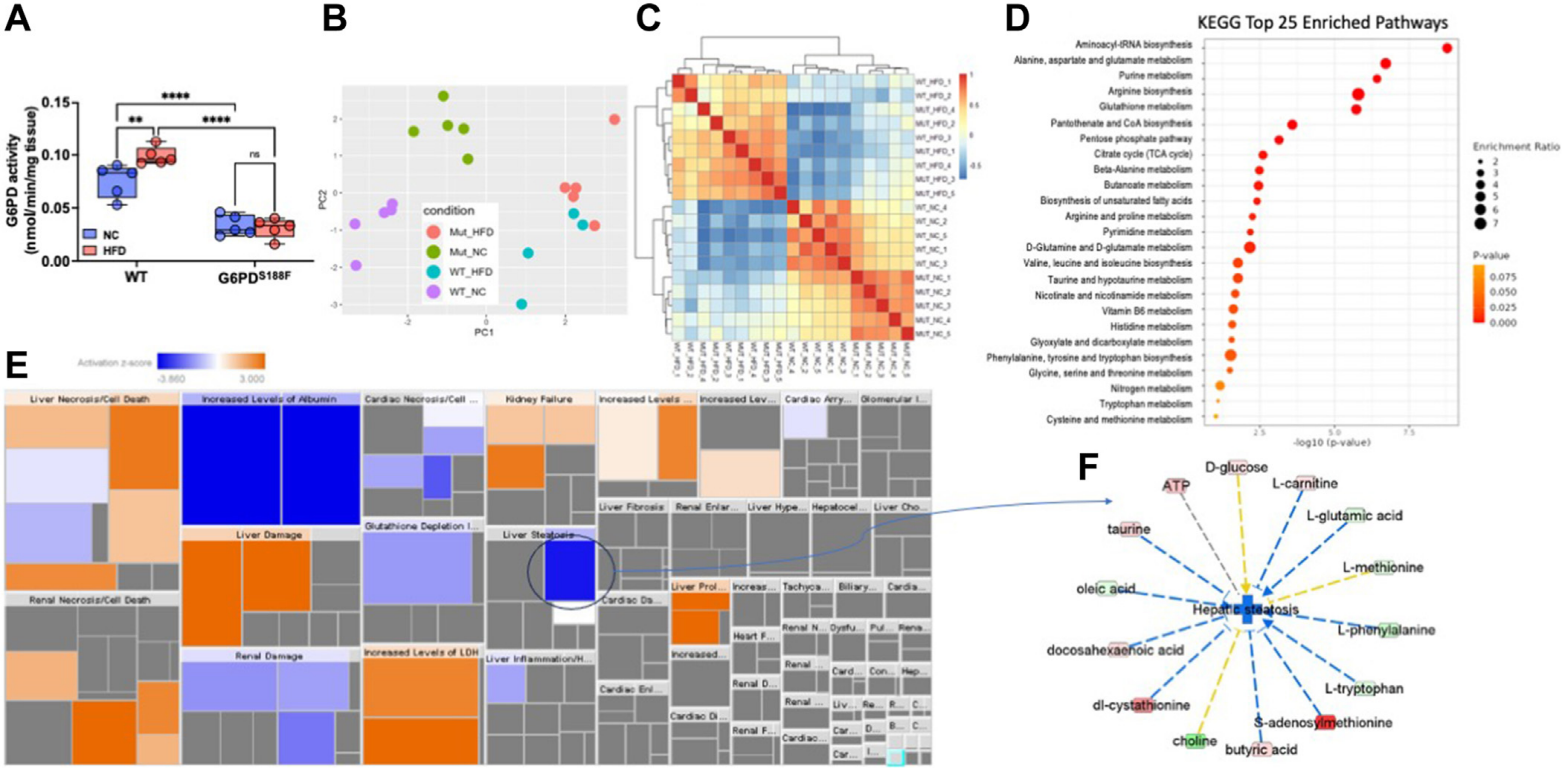
**Effect of long-term high-fat diet feeding on liver toxicity, non-alcoholic fatty liver, and circadian rhythm gene expression in the liver of wild-type and G6PD**^**S188F**^**rats.***A*, G6PD activity decreased in the liver of G6PD^S188F^ rats as compared to wild-type (WT) rats fed with normal chow (NC) and high-fat diet (HFD). *B* and *C*, PCA plot and sample correlation heat map demonstrating differential metabolism in the liver of WT and G6PD^S188F^ fed with NC and HFD. *D*, KEGG enrichment pathway analysis identified the top 25 pathways in response to G6PD mutation. *E*, IPA Tox Analysis result based on the Liver HFD metabolic dataset shows that liver steatosis is significantly (activation z-score = −1.88) inhibited in response to G6PD mutation. *F*, G6PD can regulate eight out of 14 compounds that affect hepatic steatosis as an upstream regulator. Prediction legends showing various symbols and arrows for interpretation of the IPA analysis are from Figure 2. Two-way ANOVA with *post hoc* Tukey’s multiple comparison tests was used to compare multiple groups. ∗∗*p* < 0.01 and ∗∗∗∗*p* < 0.001.

Incidences of NAFL associated with obesity are increasing worldwide (28). Therefore, we determined whether diet-induced increases in G6PD activity and metabolic reprogramming contribute to the pathogenesis of NAFL. As anticipated, G6PD activity was significantly lower in liver of G6PD^S188F^ rats as compared with wild-type rats (Fig. 4*A*). HFD increased G6PD activity in the liver of wild-type rats but not G6PD^S188F^ rats (Fig. 4*A*). Further, the principal component analysis plot and sample correlation heat map of unbiased metabolomic revealed that metabolic phenotype in the liver was different between wild-type and G6PD^S188F^ rats on NC and HFD (Fig. 4, *B* and *C*). KEGG enrichment pathway analysis suggested glycolysis; lactate; 2-hydroxyglutarate; metabolites of the PPP; 5L-glutamyl-L-glutamine pathway; indole and tryptophan pathway metabolites; acyl-C5:1; and acyl-C18:2-OH, were significantly increased in liver of HFD fed wild-type rats more than G6PD^S188F^ rats (Figs. 4*D* and S3*A*). In addition, the SMPDB enrichment pathway identified alteration of similar pathways (Fig. S3*B*). IPA function analysis predicted 15 canonical pathways were significantly and differentially changed [threshold: absolute z-score ≥2 and -log10 (*p*-value) ≥1.3] in response to G6PD mutation (Fig. S3*C*). In addition, IPA core upstream analysis predicted 29 upstream molecules were significantly and differentially modified [threshold: absolute z-score ≥2 and -log10 (*p*-value) ≥1.3] in response to G6PD mutation (Fig. S3*D*). Further, amino acid and carbohydrate metabolism-related functions were predicted to be differentially activated in response to G6PD mutation in the liver (Fig. S4). IPA network analysis identified two networks in the NC diet related to carbohydrate metabolism, inflammatory disease, immunological disease, energy production, and small molecule biochemistry, and two networks in HFD group related to immunological disease, inflammatory disease, inflammatory response (Fig. S5).

### G6PD^S188F^ variant decreases HFD-induced nonalcoholic fatty liver

**Figure 5:**
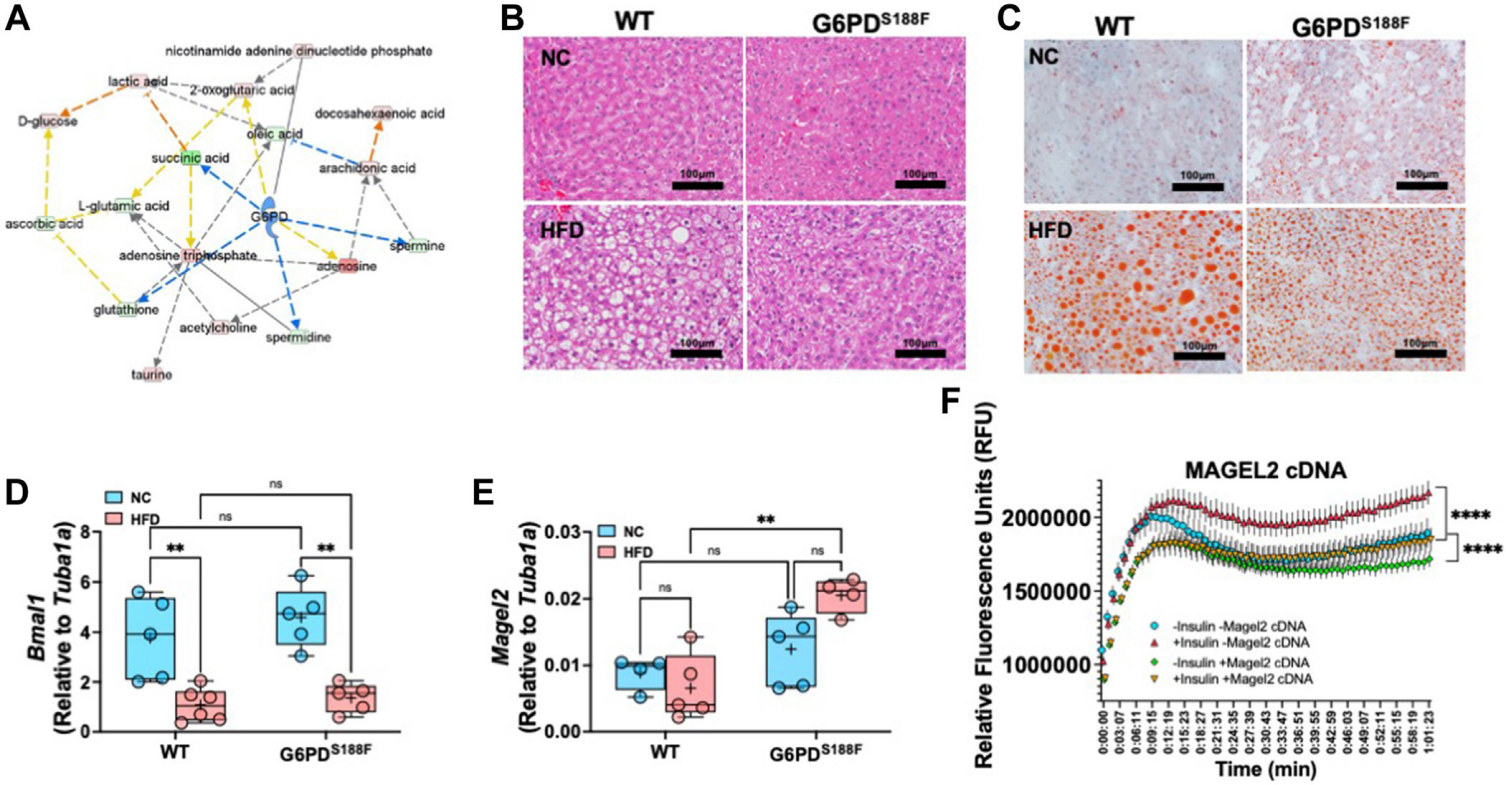
**Effect of long-term high-fat diet feeding non-alcoholic fatty liver and circadian rhythm gene expression in the liver of wild-type and G6PD**^**S188F**^**rats.***A*, G6PD can regulate metabolic pathways. Prediction legends showing various symbols and arrows for interpretation of the IPA analysis are from Figure 2. *B*, representative images of H&E staining showing lipid accumulation and fatty liver in HFD-fed wild-type (WT) rats and to a lesser extent in G6PD^S188F^ rats. Images were reused to demonstrate rigor and reproducibility in Fig. S6. *C*, representative images of Oil Red staining indicate that lipid droplets are reduced in the liver of G6PD^S188F^ rats than in wild-type (WT) rats. *D* and *E*, expression of circadian clock gene *Bmal1* decreased in the liver of both genotypes fed with HFD, while circadian clock and Prader-Willi Syndrome associated *Magel2* gene expression selectively increased in HFD-fed G6PD^S188F^ rats. *F*, overexpression of MAGEL2 significantly reduced insulin-induced fatty acid uptake and accumulation in HepG2 cells. Two-way ANOVA with *post hoc* Tukey’s multiple comparison test was used to compare multiple groups. ∗∗*p* < 0.01 and ∗∗∗∗*p* < 0.001.

While HFD feeding did not increase ALT and AST in plasma of both genotypes (Table 1), IPA Tox analysis of the liver HFD dataset shows that fatty liver disease (steatosis) was significantly [activation z-score was −1.88 and -log10 (*p*-value) was 4.06] inhibited in response to G6PD mutation (Fig. 4*E*). G6PD can regulate several compounds that affect hepatic steatosis as an upstream regulator as well as metabolic pathways (Figs. 4*F* and 5*A*). Further, histology of H&E-stained liver section revealed more lipid accumulation in the liver of HFD-fed wild-type rats than G6PD^S188F^ rats (Figs. 5*B* and S6). Similarly, Oil-Red staining showed large lipid droplets in the liver of HFD fed wild-type rats as compared with G6PD^S188F^ rats (Fig. 5*C*). Morphometric analysis and grading, based on previous recommendations (29), revealed more severe NAFL in wild-type rats [severe (50%), moderate-to-severe (17%), and moderate (33%)] than in G6PD^S188F^ rats [severe (0%), moderate-to-severe (40%), and moderate (60%)]. Downregulation of genes that encode circadian clock proteins, including BMAL1, has been associated with alteration in liver metabolism and uptake of fatty acid by hepatocytes leading to NAFL (30). Consistently, *Bmal1* expression decreased in HFD fed wild-type and G6PD^S188F^ rats (Fig. 5*D*). Although G6PD^S188F^ variant reduced the severity of NAFL, it did not rescue the expression of *Bmal1*. Instead, we found expression of *Magel2*, a gene encoding circadian clock-related protein that suppresses obesity and metabolic reprogramming associated with Prader-Willi syndrome (31), increased in HFD-fed G6PD^S188F^ rats as compared with wild-type rats (Fig. 5*E*). Along those lines, overexpression of MAGEL2 significantly reduced insulin-induced fatty acid accumulation in HepG2 cells (Fig. 5*F*).

### G6PD^S188F^ variant alters HFD-induced metabolic reprogramming in aorta

**Figure 6:**
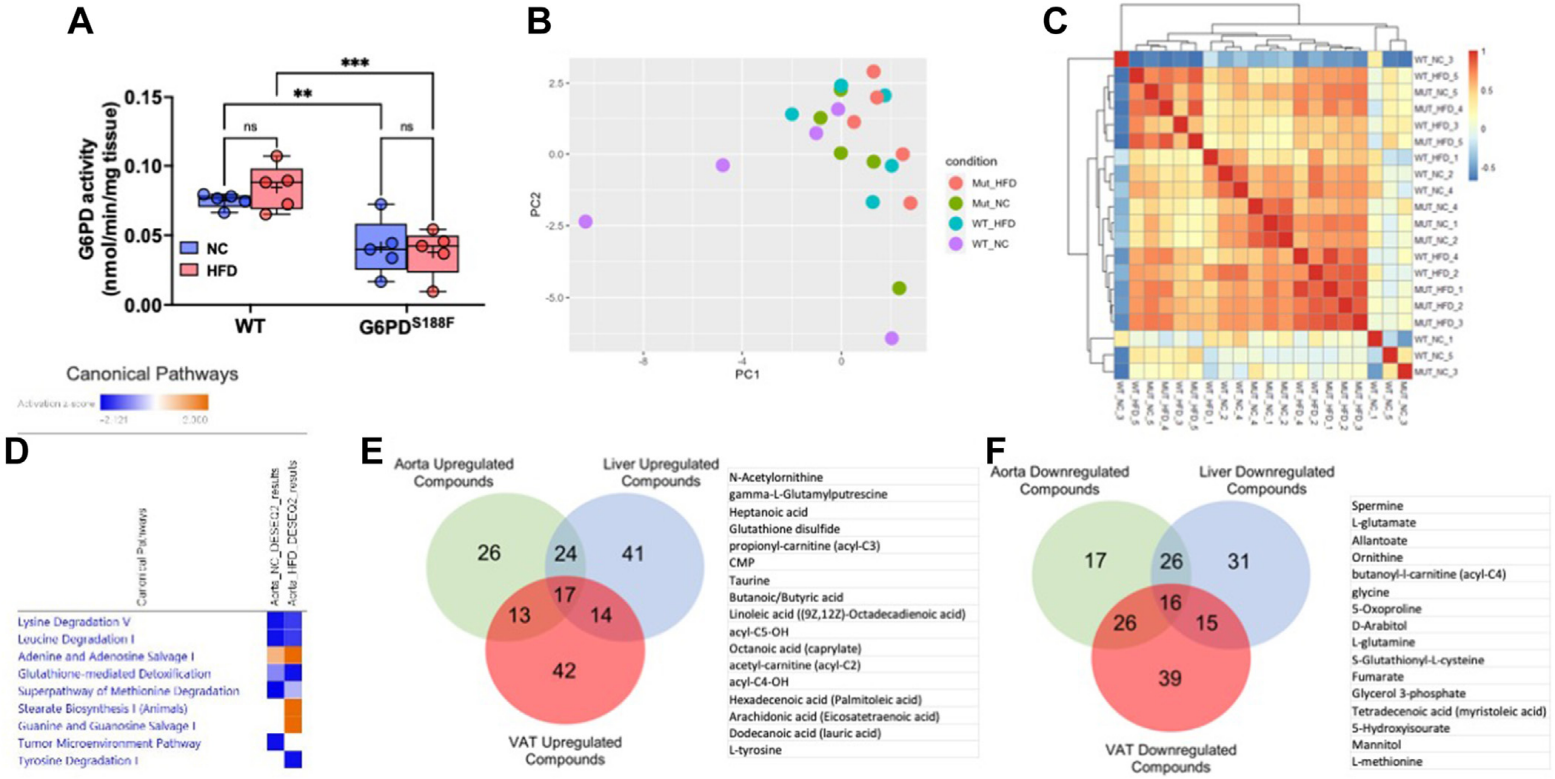
**Effect of long-term high-fat diet feeding on G6PD activity and metabolomic reprogramming in the aorta of wild-type and G6PD**^**S188F**^**rats.***A*, G6PD activity decreased in the aorta of G6PD^S188F^ rats as compared to wild-type (WT) rats fed with normal chow (NC) and high-fat diet (HFD). *B* and *C*, PCA plot and sample correlation heat map demonstrating differential metabolism in the liver of WT and G6PD^S188F^ fed with NC and HFD. *D*, IPA core analysis showing differential changes in canonical pathway function. *EandF*, venn diagram shows up (17) or down (16) regulated common metabolites in visceral adipose tissue, liver, and aorta, of HFD-fed rats. N = 5 in each group. Two-way ANOVA with *post hoc* Tukey’s multiple comparison test was used to compare multiple groups. ∗∗*p* < 0.01.

To elucidate the effect of long-term feeding of HFD on vascular tissue metabolism and function, we determined G6PD activity in the aorta of wild-type and G6PD^S188F^ rats fed with NC and HFD. G6PD activity was lower in the aorta of G6PD^S188F^ rats than in wild-type rats, and HFD did not increase G6PD activity in both genotypes (Fig. 6*A*). Principal component analysis plot and sample correlation heat map of unbiased metabolomic indicated that metabolic phenotype in the aorta partially overlapped between wild-type and G6PD^S188F^ rats on NC and HFD (Fig. 6, *B* and *C*). However, metabolites of the TCA cycle; glutathione and oxidized products of the glutathione pathway; γ-glutamyl metabolites; and carnitine and fatty acid oxidation products, increased more in the aorta of HFD-fed G6PD^S188F^ rats than wild-type rats (Fig. S7*A*). In contrast, saturated and mono/polyunsaturated fatty acids; intermediates products of the glycolytic pathway and PPP; and indole and tryptophan metabolism, decreased in the aorta of HFD-fed G6PD^S188F^ rats as compared with wild-type rats (Fig. S7*A*). Interestingly, adrenalin and dopamine, which act as vasodilators, decreased in the aorta of HFD-fed G6PD^S188F^ rats as compared with wild-type rats (Fig. S7*A*). However, sphinganine-1-phosphate (also known as dihydrosphingosine 1-phosphate), a bioactive lipid molecule implicated in biological function distinct than sphingosine-1-phosphate and in signaling that induces matrix metalloproteinase 1 (32), increased in the aorta of HFD fed G6PD^S188F^ rats as compared with wild-type rats (Fig. S7*A*). IPA function analysis predicted nine canonical pathways were significantly and differentially changed [threshold: absolute z-score ≥2 and -log10 (*p*-value) ≥1.3] in response to G6PD mutation (Fig. 6*D*). In addition, IPA core upstream analysis predicted 27 upstream molecules were significantly and differentially modified [threshold: absolute z-score ≥2 and -log10 (*p*-value) ≥1.3] in response to G6PD mutation (Fig. S7*B*). Further, IPA network analysis identified three networks in the HFD group related to small molecule biochemistry, cellular compromise, carbohydrate metabolism, energy production, lipid metabolism, immunological disease, inflammatory disease, and inflammatory response (Fig. S8).

### Identification of commonly regulated compounds in VAT, liver, and aorta samples of HFD-fed rats

We identified 17 upregulated and 16 downregulated common metabolites/compounds in VAT, liver, and aorta of HFD-fed rats (Fig. 6, *E* and *F*). KEGG and SMPDB enrichment analyses on commonly regulated compounds revealed that pathways related to glutathione metabolism, arginine biosynthesis, D-glutamine and D-glutamate metabolism, nitrogen metabolism, and beta-oxidation of very long chain fatty acids, were affected by HFD feeding (Fig. S9).

### G6PD^S188F^ variant decreases age-related, but not HFD-induced, large artery stiffness

**Figure 7:**
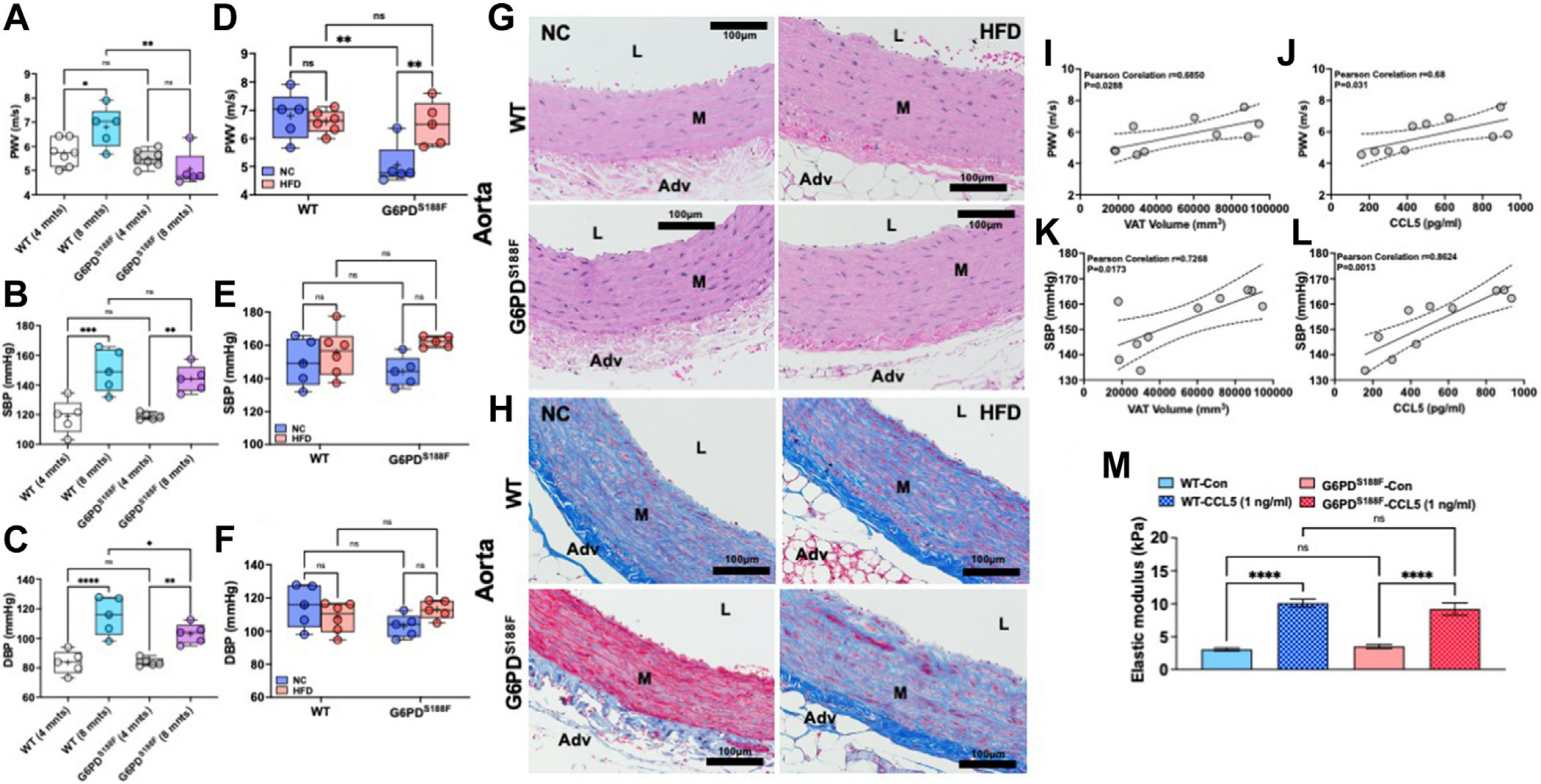
**Effect of aging and long-term high fat diet feeding on aortic stiffness and blood pressure of wild-type and G6PD**^**S188F**^**rats and VAT-derived CCL5 positively correlates with aortic stiffness and incubation of aorta with CCL5 augments elastic modulus.***A*–*C*, aging increased pulse wave velocity (PWV; index of aortic stiffness) and systolic and diastolic blood pressure in both genotypes. However, diastolic pressure in older G6PD^S188F^ was significantly lower than in age-matched wild-type (WT) rats. *D*–*F*, HFD feeding for 8 months did not increase PWV and blood pressure in older wild-type rats, and blood pressure in older G6PD^S188F^ rats, as compared with age-matched rats on a normal chow (NC) diet. However, HFD feeding increased PWV in G6PD^S188F^ rats as compared with age-matched rats on the NC diet. *G* and *H*, representative H&E and Mason’s Trichrome staining of aorta isolated from wild-type and G6PD^S188F^ rats fed with high-fat diet (HFD) or normal chow (NC) is shown. H&E staining images show hypertrophy of the medial layer in HFD-fed rats and Manson’s Trichrome staining shows collagen and fibrosis of the aorta is less pronounced in G6PD^S188F^ rats as compared with wild-type rats on NC but not on long-term HFD. Images were reused to demonstrate rigor and reproducibility in Fig. S10. *I* and *J*, Pearson’s correlation shows a positive correlation between visceral adipose tissue (VAT) volume-systolic blood pressure (SBP) and -pulse wave velocity (PWV). *K* and *L*, Pearson’s correlation shows a positive correlation between VAT-derived CCL5-SBP and -PWV in G6PD^S188F^ rats but not wild-type rats. *M*, isolated aorta from wild-type and G6PD^S188F^ rats was incubated with CCL5 (1 ng/ml) *ex vivo* and after 72 h elastic modulus was determined by atomic force microscopy. Application of CCL5 increased elastic modulus. Two-way ANOVA with *post hoc* Tukey’s multiple comparison test were used to compare multiple groups. ∗*p* < 0.05; ∗∗*p* < 0.01; ∗∗∗*p* < 0.005; and ∗∗∗∗*p* < 0.001.

Obesity and metabolic reprogramming have been implicated in the pathogenesis of vascular remodeling and diseases (33, 34). Since we embarked to study the effects of long-term feeding of HFD on vascular function, we first determined age-related (4 months *versus* 8 months) changes in vascular function. To evaluate the effects of aging on vascular function, we performed echocardiography and catheterization on 4-month and 8-month-old wild-type rats fed with NC diet. Large artery stiffness increases with age (8, 11). Consistently, pulse wave velocity (PWV), a functional parameter influenced by arterial wall stiffness that proportionately increases with stiffening of arteries (35, 36), increased significantly with aging in wild-type rats (Fig. 7*A*). Notably, PWV did not increase in aging G6PD^S188F^ rats (Fig. 7*A*). Also, we found that systolic and diastolic blood pressures (SBP and DBP, respectively) increased in wild-type rats, but the increase in DBP was significantly lower in G6PD^S188F^ than in wild-type rats (Fig. 7, *B* and *C*). These results indicated that changes in vascular characteristics with aging were different between wild-type and G6PD^S188F^.

Next, since sphinganine-1-phosphate—which has an opposite role than sphingosine-1-phosphate in the regulation of TGFβ-signaling and TGFβ-induced fibrosis (32)—increased in the aorta of HFD-fed G6PD^S188F^ rats, we predicted that G6PD^S188F^ variant may reduce long-term HFD feeding-induced arterial stiffness. Unexpectedly, PWV slightly increased (*p* < 0.05) in G6PD^S188F^ rats fed with HFD as compared with NC (Fig. 7*D*). HFD feeding did not increase PWV and blood pressure in wild-type rats (Fig. 7, *E* and *F*).

### Age-related, but not HFD-induced, collagen expression is less in the aorta of G6PD^S188F^ variant rats than in wild-type rats

To determine whether fibrosis is the cause of arterial stiffness associated with aging and HFD feeding, we performed a histological evaluation of the aorta. Morphometric analysis performed on aorta after H&E staining shows increased hypertrophy (media-to-lumen ratio) of aorta from HFD fed wild-type rats (Fig. 7*G*; WT+NC: 0.095 ± 0.004 and WT+HFD: 0.117 ± 0.009; *p* < 0.05), but not HFD fed G6PD^S188F^ (Fig. 7*G*; G6PD^S188F^+NC: 0.104 ± 0.007 and G6PD^S188F^+HFD: 0.091 ± 0.008; NS). Furthermore, Masson’s Trichrome staining showed a clear difference in the expression of collagen (blue staining)—an indicator of arterial fibrosis—in G6PD^S188F^ variant rats as compared to wild-type rats. Remarkably, collagen (blue staining) was lesser, and fibrosis was not advanced, in the medial layers of the aorta of 8-month-old G6PD^S188F^ rats as compared to their age-matched wild-type littermates on NC diet (Fig. 7*H*), but collagen staining increased and fibrosis advanced in medial layers of aorta of HFD fed 8 months G6PD^S188F^ rats (Figs. 7*H* and S10*A*). In wild-type rats, aortic fibrosis between HFD and NC groups was not different (Fig. 7*H*). Similarly, while H&E staining of the coronary artery was not different in both genotypes fed with HFD or NC (Fig. S10*B*), Masson’s Trichrome staining showed advanced perivascular collagen staining (indicator of fibrosis) in both genotypes, but slightly more in wild-type rats than G6PD^S188F^ rats, fed with HFD compared to NC (Fig. S10*C*).

### Expression of smooth muscle cell differentiation-related genes was not altered by HFD feeding in the aorta of G6PD^S188F^ and wild-type rats

Dedifferentiated smooth muscle cell (SMC) phenotype contributes to the production of extracellular matrix proteins in the vessel wall (37). Because HFD increased collagen (extracellular matrix proteins and a marker of dedifferentiation) in the aorta, we determined the expression of SMC differentiation phenotype marker genes in the aorta of wild-type and G6PD^S188F^ rats fed with NC and HFD. Expression of SMC differentiation marker genes: myocardin (*Myocd*), myosin heavy chain 11 (*Myh11*), and leiomodin 1 (*Lmod1*), did not decrease in the aorta of wild-type and G6PD^S188F^ rats fed with HFD as compared to NC diet (Fig. S11*A*). Conversely, expression of *Klf4*, a transcription factor that promotes dedifferentiation (synthetic phenotype) of SMC (37), did not increase in the aorta of wild-type and G6PD^S188F^ rats fed with HFD (Fig. S11*A*). Besides, increased collagen/fibrosis of the vessel wall, calcification of SMC contributes to the development of large artery stiffness (38). Consistently, while expression of muscle segment homeobox-1 (*Msx1*), a transcription factor that evokes osteogenic gene expression, increased in aorta of G6PD^S188F^+HFD as compared to G6PD^S188F^+NC rats (Fig. S11*B*), *Msx2* and *Runx2* or *Epha4*—other osteogenic/calcification-related genes—did not change in aorta of wild-type and G6PD^S188F^ rats (Fig. S11*B*). *Msx1* and *Msx2* transcription factors are involved in osteogenic gene expression and promoting shear-induced inflammation in endothelial cells (39, 40, 41).

### HFD-induced large artery stiffness and blood pressure positively correlates with VAT and VAT-derived CCL5 in G6PD^S188F^ variant rats, and CCL5 induces stiffness of aorta from both genotypes

Since we did not find that HFD evoked SMC dedifferentiation and changes in SMC phenotype contributed to HFD-induced increase of collagen and extracellular matrix remodeling, we speculated that SMC-independent or extra-vascular factor(s) perhaps contributed to extracellular matrix fibrosis and to stiffening of the aorta. In that context, perivascular VAT and NAFL have been shown to increase arterial contraction and stiffness, respectively (5, 7). While HFD-induced NAFL decreased (Fig. 5, *B* and *C*), aortic stiffness increased (Fig. 7*D*), in G6PD^S188F^ rats. This indicated that inter-organ communication between NAFL and large arteries was perhaps not responsible for evoking aortic fibrosis and stiffness. Therefore, we postulated that increased perivascular VAT and/or VAT-derived mediators, such as inflammatory cytokines, potentially induced aortic stiffening. Interestingly, SBP and PWV significantly correlated with VAT (Fig. 7, *I* and *K*). Similarly, SBP and PWV showed significant positive correlations with increased VAT-derived CCL5 (Fig. 7, *J* and *L*). This indicated that elevated VAT-derived CCL5 potentially induced aortic stiffness. Therefore, to test this hypothesis, we isolated aorta from wild-type and G6PD^S188F^ rats and incubated them *ex vivo* with CCL5 (1 ng/ml) for 72 h and then determined stiffness by AFM as described previously (42, 43). Remarkably, the application of CCL5 to the aorta of both genotypes increased stiffness (elastic modulus; Fig. 7*M*).

## Discussion

Considering diet-induced obesity-associated serious health issues are emerging globally, we conducted this study to determine the consequence of HFD-induced obesity on metabolic reprogramming and multi-organ diseases. In addition, our goal was to determine a role of G6PD, the key rate-limiting enzyme in the PPP that is induced and activated by hormones (insulin) and diet or oxidative stress (16), in the pathogenesis of HFD-induced obesity, metabolic reprogramming, and multi-organ diseases, using a novel rat model of loss-of-function Mediterranean G6PD variant generated with CRISPR-editing. Our novel findings suggested that long-term (8 months) HFD feeding increased body weight, elicited metabolic reprogramming in multiple organs, augmented accumulation of visceral fat, and induced inflammation of VAT and NAFL. Interestingly, loss-of-function G6PD^S188F^ variant, reduced HFD-induced weight gain, adipocyte hypertrophy, and NAFL. Although the G6PD^S188F^ variant significantly reduced age-related aortic stiffening, paradoxically HFD-induced aortic stiffness increased in G6PD^S188F^ rats. Furthermore, our novel findings suggest that inter-organ communication between perivascular VAT and arterial tissue, *via* increased VAT-derived CCL5, is responsible for eliciting aortic stiffness in both genotypes.

HFD feeding rat/mouse models are widely used to determine the consequences of diet-induced obesity on dyslipidemia, adipogenesis, NAFL, and vascular diseases. In the present study, our results indicate that HFD elicited metabolic reprogramming in multiple organs and caused dyslipidemia but did not increase blood glucose in rats. HFD increased G6PD activity in VAT. G6PD, a member of the family of lipogenic enzymes responsible for fatty acid synthases and metabolism, is induced in adipose tissue and liver by diet and is overexpressed in obese and diabetic Zucker (fa/fa) rats, *db/db* and *ob/ob* mice, and diet-induced obese mice (16, 44, 45). Concurrently, unbiased metabolomic analysis revealed metabolites of G6PD- and redox-dependent pathways including ribose-1-phosphate; γ-glutamyl metabolites; carnitines (acyl-C14:1, acyl-C18, and acyl-C20:4); spermine; methionine-s-oxide; and indole-3-acetate; increased in HFD fed/obese rats. In this context, G6PD^S188F^ rats, which had less G6PD activity in VAT, the metabolites of the PPP were decreased, and G6PD^S188F^ variant moderated HFD-induced reprogramming of carnitine and fatty, γ-glutamyl, polyamine, sulfur, and indole metabolism. Conversely, GSSG levels were significantly increased in the VAT of G6PD^S188F^ rats. Altogether, these results indicated increased G6PD activity perhaps contributed to metabolic reprogramming in adipose tissue of HFD-fed/obese rats.

Metabolic reprogramming or imbalance of metabolic homeostasis activates resident immune cells and evokes inflammation of adipose tissue (46). Consistently, HFD increased pro-inflammatory chemokines in VAT. We found that HFD-induced synthesis of CCL2 significantly increased in VAT of wild-type but not G6PD^S188F^ rats. Further, other inflammatory chemokines (CCL3, CCL5, and CCL7) increased in VAT of HFD-fed wild-type and G6PD^S188F^ rats. Although the increase in CCL7 was significantly less in VAT of G6PD^S188F^ rats than wild-type rats. Since G6PD^S188F^ variant attenuated HFD-induced CCL2 and CCL7 increase and reduced CXCL2 and G-CSF levels in VAT, this suggests that the G6PD^S188F^ variant, at least partly, moderated infiltration of polymorphonuclear leukocytes and differentiation of precursor stem cells to mature granulocytes leading to inflammation in VAT tissue. In this context, other studies have reported that overexpression of G6PD evokes an inflammatory response in the adipose tissue of diabetic mice (27), and conversely, G6PD deficiency reduces diet-induced CCL2 and inflammation of adipose tissue in mice (47). Unexpectedly, we also found HFD feeding significantly decreased 15(S)-HEPE – a bioactive oxylipin that suppresses leukotriene B4-induced chemotaxis of leukocytes (25, 26) – in VAT. Therefore, we propose decreased 15(S)-HEPE contributed to inflammation of adipose tissue in both genotypes fed with HFD. Nonetheless, our findings suggest G6PD deficiency in G6PD^S188F^ rat, at least partly, lessened inflammation of adipose tissue and decreased adipocyte size in HFD-induced obesity model.

Next, we found significantly smaller adipocytes in VAT of G6PD^S188F^ rats than wild-type rats fed with NC and HFD. This is perhaps because G6PD deficiency impaired G6PD-derived NADPH-dependent lipogenic activity and fatty acid synthesis in VAT of G6PD^S188F^ rats as compared to wild-type rats. Inflammation of adipocytes causes the resident immune system to release increased amounts of mediators that triggers excess influx of lipids and glucose resulting in enhanced adipocyte hypertrophy (46). Therefore, we suggest HFD-induced inflammatory chemokines increased adipocyte size and G6PD deficiency lessened CCL2/CCL7-mediated immune response potentially reduced adipocyte hypertrophy in HFD-fed G6PD^S188F^ rats.

Obesity-associated NAFL is a major health issue with inadequate treatment. In the US, it affects approximately 30% of the general population (48). It is believed that an increased influx of metabolic substrates, like glucose and fatty acids, in hepatocytes together with altered metabolism causes NAFL (28). G6PD activity increased in the liver of wild-type rats fed with HFD, and the activity of the PPP and glycolytic pathway alongside G6PD-derived NADPH-dependent indole metabolism as well as carnitine and fatty acid metabolism was augmented by HFD feeding. In addition, 2-hydroxyglutarate, an alternative carboxylic acid that increases after impaired mitochondrial function and metabolic reprogramming (49), and stearic and myristoleic acids, increased in the liver of HFD-fed obese wild-type rats. 2-Hydroxyglutarate increased in the liver of patients with NASH, a severe form of NAFL, positively correlates with increased AST enzyme and is predictive of future NAFL-related issues (50, 51, 52). Since increased 2-hydroxyglutarate contributes to the development of oxidative stress inhibits chromatin-modifying enzymes and mTOR in cancer (53) and elicits inflammation (49), we predict 2-hydroxyglutatrate-associated oxidative stress and inhibition of epigenetic enzymes potentially contribute to the progression of HFD-induced NAFL. Besides, fatty acid overload is an important cause of NAFL development (28). Interestingly, our results revealed that G6PD^S188F^ variant, with reduced G6PD activity, prevented metabolic reprogramming, reduced 2-hydroxyglutatrate and fatty acid overload, and decreased the severity of NAFL.

In pursuit of determining the mechanism of HFD-induced NAFL led to the finding that HFD feeding decreased the circadian clock-related gene, *Bmal1*, in both genotypes. Desynchrony of circadian rhythms are connected to metabolic disturbances (such as altered lipid, glucose, and cholesterol metabolism) in NAFL disease (30). Although G6PD^S188F^ variant did not prevent the loss of HFD-induced *Bmal1* expression, intriguingly it increased *Magel2*, a circadian clock-controlled gene whose disruption results in some of the characteristics of Prader-Willi Syndrome (31, 54). Recently, we found that *Magel2* gene is hypomethylated and is increased in the vascular tissue of G6PD^S188F^ rats (55). MAGEL2 modulates the ubiquitination of CRY1, which is also regulated by heavy metals (56), and possibly plays a role in regulating the circadian clock (57) and *Magel2* knockout increases adiposity and alters metabolism in mice (31, 54). Conversely, since MAGEL2 overexpression decreased insulin-induced fatty acid/lipid accumulation in HepG2 cells, we propose G6PD^S188F^ variant-mediated increase in *Magel2*/MAGEL2 expression, at least partially, played a role in reducing the severity of HFD-induced NAFL. However, further studies are needed to elucidate this plausibility.

Reduced fatty liver and total cholesterol indicate that lipid accumulation and/or synthesis was potentially attenuated in the liver of HFD-fed G6PD^S188F^ variant rats. It is well established that G6PD-derived NADPH contributes to the synthesis of cholesterol and hence it is reasonable to assume that the loss-of-function G6PD^S188F^ variant reduced cholesterol synthesis in the liver and lowered total cholesterol levels in the serum of G6PD^S188F^ variant rats. However, elevated LDL and decreased HDL suggest that lower total cholesterol perhaps altered cholesterol homeostasis in HFD-fed G6PD^S188F^ rats. The liver is the major site for cholesterol homeostasis maintenance. VLDL produced in the liver is converted to LDL by the removal of the triacylglycerol. LDL carries cholesterol to the peripheral tissues and organs where it is used for various cellular transcations including building cell walls and maintaining cell wall integrity. While HDL removes cholesterol from the peripheral organs and transports it to the liver. Although increased LDL and decreased HDL are risk factors for vascular diseases, vascular pathology was not exacerbated in HFD-fed G6PD^S188F^ rats as compared with the wild-type rats. Therefore, we propose alterations in LDL and HDL levels were perhaps adaptive compensatory changes to maintain the supply of cholesterol from the liver to the peripheral tissues and to reduce the removal of cholesterol from tissues.

Obesity is a critical and independent risk factor for vascular diseases (58). It contributes to the pathogenesis of hypertension, large artery stiffness, and atherosclerosis (10, 58). Large artery stiffening, also known as loss of Windkessel function, impairs the cushioning function, which protects the microvasculature from potentially harmful fluctuations in pressure and blood flow and exerts widespread detrimental effects on organ function (8). It induces isolated systolic hypertension, abnormal ventricular-arterial interactions that promote heart failure, and end-organ damage (8, 9, 10). Large artery stiffness is a progressive disease that increases with age (8). Therefore, we first determined the effect of aging on arterial stiffness and hypertension in NC-fed wild-type and G6PD^S188F^ variant rats. Our results showed that stiffness and hypertension increased with aging in wild-type rats. Consistently, others have shown arteries of older as compared to younger animals express more collagen and are stiffer (11). In some cases, large artery stiffness causes isolated systolic hypertension which is characterized by increased SBP with normal or low DBP (*i.e.*, increased pulse pressure). In our study, PWV, SBP, and DBP increased in wild-type rats with aging. These findings are consistent with a study that suggests DBP positively correlates with stiffness and DBP is a stronger predictor of increases in arterial stiffening (59). In contrast, PWV did not increase in G6PD^S188F^ rats with aging, and it was lower in 8-month-old G6PD^S188F^ rats as compared to their wild-type littermates. However, SBP and DBP increased with increasing age of G6PD^S188F^ rats but not as much as in their wild-type littermates. Previously, we have shown that inhibiting G6PD activity or silencing G6PD expression reduces vascular tone and blood pressure (60, 61), and others have shown that G6PD deficiency prevents angiotensin-induced hypertension and atherosclerosis in mice (19, 20). Our current findings demonstrate that G6PD^S188F^ rats, which have reduced G6PD activity, moderated the age-related collagen expression/fibrosis and stiffening of large arteries and further supported the notion that the G6PD^S188F^ variant protects from certain, if not all, types of vascular diseases.

Next, we determined the effect of long-term HFD feeding on aortic stiffness and hypertension. Interestingly, in wild-type rats, HFD feeding did not increase stiffness. The most likely interpretation of this result is that perhaps HFD did not worsen the stiffness in older rats because of already augmented stiffness and aortic fibrosis due to aging. However, HFD feeding slightly increased stiffness in G6PD^S188F^ variant rats, in which age-related stiffness and aortic fibrosis were less severe than in their age-matched wild-type littermates. Since augmented glutathione, reductive stress that promotes protein aggregation (62), and dihydroascorbate, an oxidation product of ascorbic acid associated with increased oxidative stress, increased in the aorta of HFD-fed G6PD^S188F^ rats; we suggest increased oxidative-reductive stress, which is implicated in the pathogenesis of fibrosis and arterial stiffness (9), in the vessel wall most probably contributed to increasing aortic stiffness in HFD fed G6PD^S188F^ rats.

Besides evoking metabolic reprogramming, HFD increased the expression of *Msx-1*, a transcription factor, that activates the expression of osteogenic genes (40) and promotes osteogenic differentiation and inflammation of endothelial cells during the remodeling of collateral arteries (40, 41). Since SMC-specific ablation of *Msx1* and *Msx2* attenuates atherosclerotic calcification and aortic stiffness in diabetic mice (63), we suggest increased MSX1, at least partly, contributed to stiffening of aorta in HFD-fed G6PD^S188F^ rats. HFD feeding did not modify the expression of genes that encode SMC differentiation proteins in the aorta of both genotypes. These findings were unexpected because our previous studies showed that G6PD^S188F^ variant increases SMC differentiation proteins and reduces short-term (4 months) HFD feeding-induced arterial stiffness and hypertension (21). Therefore, from these results, we inferred that in addition to metabolic reprogramming and gene expression changes within the vascular wall cells, other extra-vascular factors, potentially contributed to the increase aortic stiffness of long-term (8-months) HFD-fed G6PD^S188F^ rats.

Because previous studies imply that NAFL and perivascular VAT, respectively, increase arterial stiffness (7) and contraction (5), we speculated that mediators derived from either HFD-induced NAFL or increased perivascular VAT contributed to the pathogenesis of aortic stiffness. However, since the severity of HFD-induced NAFL decreased and paradoxically aortic stiffness increased in G6PD^S188F^ variant rats, we eliminated the possibility of NAFL or NAFL-derived factors as contributors of aortic stiffening. Unexpectedly, we found a positive correlation between increased stiffness and VAT as well as VAT-derived CCL5. This suggested that VAT-derived CCL5 potentially contributed to aortic stiffening. To confirm this hypothesis, we incubated aorta isolated from wild-type and G6PD^S188F^ variant rats, with CCL5 for 72 h and then determined elastic modulus (indicator of stiffness) by AFM. Intriguingly, CCL5 increased the elastic modulus of the aorta from both genotypes. These findings imply that inflammation of perivascular VAT turned into maladaptive inter-organ signal that contributed to evoke aortic stiffness.

**Figure 8:**
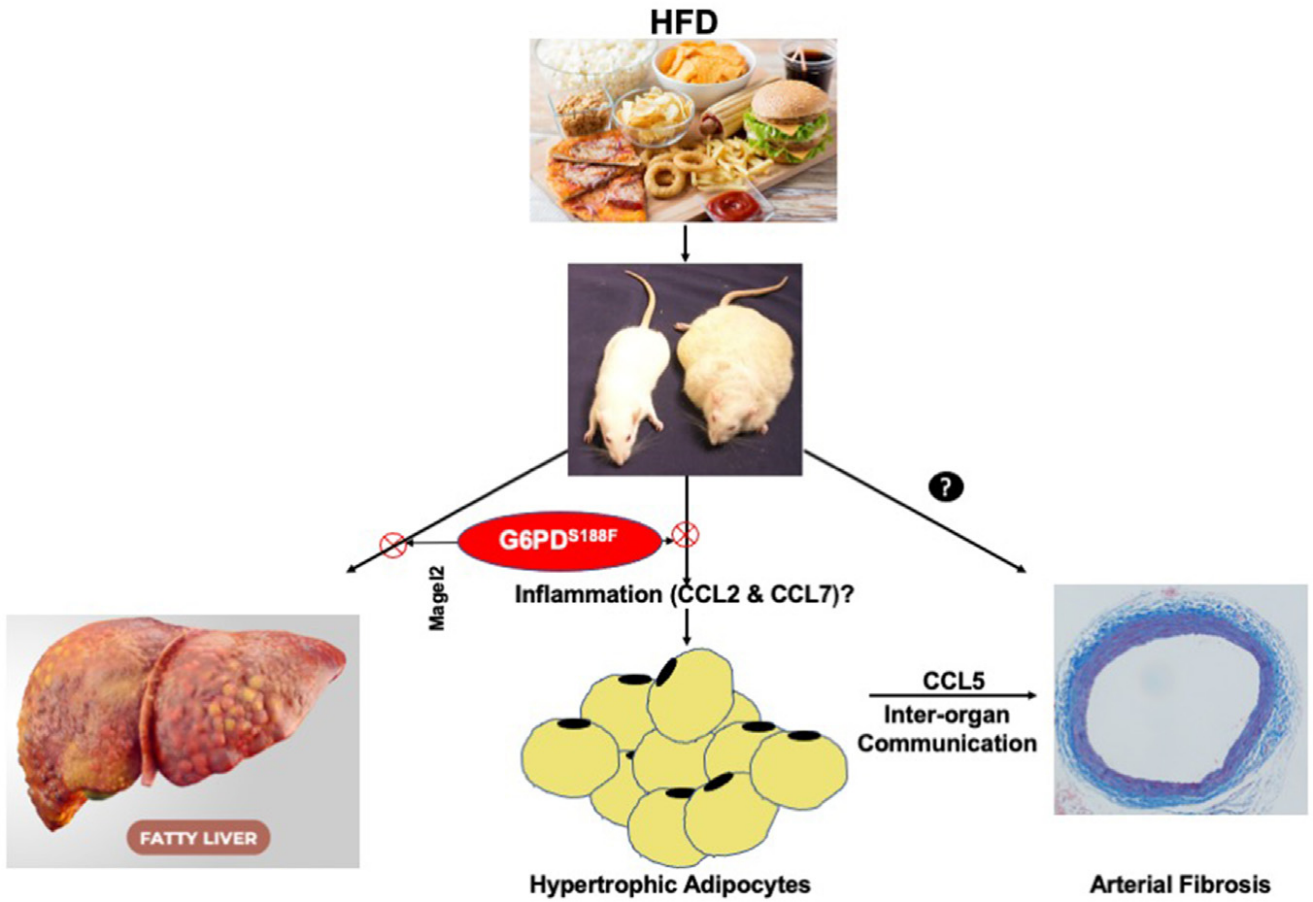
**Schematic illustration of key findings.** Long-term (8 months) high-fat diet (HFD) feeding increased body weight in wild-type rats more than G6PD^S188F^ rats. In addition, in G6PD^S188F^ rats, HFD-induced non-alcoholic fatty liver (fatty liver) and adipocyte hypertrophy (growth) were reduced as compared to wild-type rats, respectively, by presumably increasing *Magel2* and reducing inflammation (CCL2 and CCL7). Moreover, we found HFD-elicited maladaptive inter-organ communication between adipose tissue and aorta *via* CCL5 that mediated aortic fibrosis and increased aortic stiffness.

In summary, our findings demonstrated that HFD feeding induced metabolic reprogramming in multiple organs potentially contributed to increased body weight, adipocyte hypertrophy and inflammation, NAFL, and aortic stiffening. Furthermore, our findings uncovered, for the time being, that inter-organ communication such as adipose tissue-derived CCL5 mediates HFD-induced aortic stiffening (Fig. 8). Finally, our findings established that G6PD^S188F^ mutation, at least partially, moderated HFD-induced obesity, adipocyte hypertrophy, and fatty liver (Fig. 8).

## Experimental procedures

Detailed methods are available in the Online Data Supplement.

### Animal models and experimental protocols

All animal experiments were approved by the New York Medical College Animal Care and Use Committee and all procedures conformed to the guidelines from the NIH Guide for the Care and Use of Laboratory Animals. G6PD^S188F^ variant rats were generated in our laboratory using CRISPR editing methods (21). Eight-to-10 week old male rats G6PD^S188F^ and their wild-type (WT) littermates were randomly divided into two groups and one group was fed normal chow (NC, containing: 24.1% protein; 6.4% fat; and 54.4% carbohydrate; 13.6% fat of total calories; 5001; LabDiet), while second group was fed high fat (containing: 20% protein; 60% fat; and 20% carbohydrate; 60% fat of total calories; D12492, Research Diets, Inc) diet for 8 months (32 weeks). All rats were weighed every 8 weeks. In this study, we made comparisons with age-matched rats, which were fed an NC diet. All rats were anesthetized with inhalation of Isoflurane (isoflurane, USP; 1-chloro-2,2,2-trifluoroethyl difluoromethyl ether; induced at 5% and maintained at 1.5%) and placed on a heated table. Echocardiography, hemodynamic measurements, and micro-CT were performed as described in the Online Data Supplement.

### Statistical analysis

Graphs and statistical analyses were prepared with GraphPad Prism 9.2 (GraphPad Software, Inc) and MetaboAnalyst 4.0. Normal distribution was determined by normality and lognormality test and outliers were identified by ROUT test. Data are presented as the Box and Whisker plot or bar graphs with mean ± SD of the number of samples (n) from different animals. Two-way ANOVA with *post hoc* Tukey’s multiple comparison tests was used to compare multiple groups. Values of *p < 0.05* were considered significant.

## Data availability

All data supporting the findings of this study are described below and in Online Data Supplement. Orginal data is available upon request.

## Supporting information

This article contains supporting information.

## Conflict of interest

The authors declare that they have no conflicts of interest with the contents of this article.
