# Supplementary material for: Loss-of-function G6PD variant moderated high-fat diet-induced obesity, adipocyte hypertrophy, and fatty liver in male rats
Source: J Biol Chem. 2024 Jun 12;300(7):107460. doi: 10.1016/j.jbc.2024.107460 (PMC11328872; doi:10.1016/j.jbc.2024.107460)
Supplement: Supporting Information [file mmc1.docx]

***Supplement***

**Loss-of-function Mediterranean G6PD variant moderated high fat diet-induced obesity, adipocyte hypertrophy, and fatty liver in male rats**

Shun Matsumura,^1^ Christina Signoretti,^1^ Samuel Fatehi,^1^ Bat Ider Tumenbayar,^2^ Catherine D’Addario,^1^ Erik Nimmer,^3^ Colin Thomas,^3^ Trisha Viswanathan,^1^ Alexandra Wolf,^1^ Victor Garcia,^1^ Petra Rocic,^4^ Yongho Bae,^3,5^ S M Shafiqul Alam,^6^ Sachin A. Gupte^1*^

^1^Department of Pharmacology, New York Medical College, Valhalla, NY 10595

^2^Department of Pharmacology and Toxicology, Jacobs School of Medicine and Biomedical Sciences, University at Buffalo, State University of New York, Buffalo, NY 14203

^3^Department of Biomedical Engineering, School of Engineering and Applied Sciences, University at Buffalo, State University of New York, Buffalo, NY 14260

^4^Department of Physiology & Pharmacology, SHSU College of Osteopathic Medicine, Conroe, TX 77304

^5^Department of Pathology and Anatomical Sciences, Jacobs School of Medicine and Biomedical Sciences, University at Buffalo, State University of New York, Buffalo, NY 14203

^6^Department of Pathology, Microbiology, and Immunology (PMI), New York Medical College, Valhalla, NY, USA, 10595

**Short Title**: G6PD^S188F^ variant and HFD

**Address Correspondence to**:

Sachin A. Gupte

Department of Pharmacology

BSB 519B, New York Medical College

15 Dana Road, Valhalla, NY 10595

Tel: 914-309-7529

Email: [s_gupte@nymc.edu](mailto:s_gupte@nymc.edu) or [sachin_gupte@yahoo.com](mailto:sachin_gupte@yahoo.com)

**Methods:**

***Micro-CT:*** Micro-CT (Quantum GX2 microCT Imaging System, PerkinElmer, Inc., USA) was performed at month 0, month 2 and month 8. It was performed under anesthesia with 1-2% isoflurane and scanned whole body. We analyzed volume of visceral adipose tissue (VAT) and subcutaneous adipose tissue (SAT) using Analyze 14.0 (AnalyzeDirect, Inc) software.

***Echocardiography:*** Echocardiography was performed in 1-2% isoflurane-anesthetized rats using a Vevo 770 imaging system (VisualSonics, Toronto, ON, Canada). Briefly, at the end of the experiment, two-dimensional parasternal short axis views were obtained. M-Mode assessment of LV function was performed, and LV parameters were measured as described previously. Pulse wave velocity (PWV) was determined from transit time between Doppler flow signals in the carotid and iliac arteries. Doppler signals and electrocardiogram (ECG) were recorded simultaneously, and the data were stored for subsequent off-line analysis. The pulse-transit time from the carotid to iliac arteries (T) was calculated by subtracting the R-carotid foot time interval from the mean R-iliac foot time interval. The distance (D) between the points of probe applanation over the carotid and iliac arteries was measured using a tape measure. PWV was calculated as: PWV = Distance (D)/Time (T). The carotid peak velocity was measured with pulsed wave Doppler.

***Hemodynamics:*** At the end of the experimental protocol, rats were anesthetized using 5% isoflurane, after which 1-2% isoflurane was used to maintain anesthesia during the entire duration of the surgery and data acquisition. Body temperature of the animal during the surgery was maintained using a heating pad. About 3 cm^2^ of skin over the ventral neck region was exposed to locate the right common carotid artery. After carefully isolating the artery, a 1.4 F Millar Micro-Tip pressure catheter was inserted into the right common carotid artery and systolic blood pressure was measured. Data were acquired and analyzed using PowerLab (ADI Instruments).

***H&E staining of aorta, coronary artery, and liver cross section*:** Aorta, coronary artery, VAT, and liver was fixed in 10% NBF and was blocked and embedded in paraffin, after which 5-µm sections were cut and stained with hematoxylin/eosin.

***Oil red staining of liver cross section*:** Frozen sections of liver samples were stained with Oil Red O. Briefly, frozen sections were placed in 10% Formalin for 2-5 minutes then rinse slide in tap water. The sections were place in Propylene glycol for 2 minutes, incubated in pre-hearted Oil Red O stain at 60°C for 6 mins excess stain was removed by washing in water 2-4 times, and mounted aqueous mounting media.

***MAGEL2 over-expression in HepG2 cells and fatty acid uptake and accumulation:*** For fatty acid uptake assays, the human hepatocyte cell line, HepG2 (ATCC, HB-8065), was used. Cells were cultured with Dulbecco's Modified Eagle Medium (DMEM) (GIBCO, 11965092), containing 10% fetal bovine serum (R&D Systems, S11150H), penicillin-streptomycin (GIBCO, #15070063) and L-glutamine (GIBCO, #A2916801). To overexpress MAGEL2, HepG2 cells were grown in 96 well plates and transfected with MAGEL2 lentiviral activation particles, which is a SAM transcription activation system designed to specifically upregulate expression of the Magel2 gene via lentiviral transduction (Santa Cruz Biotechnology, USA). MAGEL2 lentiviral activation particles contains a deactivated Cas9 (dCas9) nuclease (D10A and N863A) fused to the transactivation domain VP64, an MS2-p65-HSF1 fusion protein, and a target-specific 20 nt guide RNA that provides a robust transcription activation system to upregulate MAGEL2. HepG2 cells were transfected with lentivirus particles (2.5x10^3^ pfu) for 48 hours, following manufacturer’s protocol. After transfection, cells were washed with Hanks’s Balanced Salt Solution (+ Calcium Chloride and Magnesium Chloride) (HBSS) (GIBCO, 14025092) and starved for 30min in fresh HBSS at 37°C. After starvation, the HBSS was removed, and cells were either pre-treated with HBSS or 100nM of insulin from bovine pancreas (Sigma-Aldrich, I5500), prepared in HBSS, for 15 min. Prior to fatty acid uptake analysis, the QBT fatty acid uptake (Molecular Devices, R8132) dye was dissolved in HBSS containing 0.2% fatty acid free bovine serum albumin (BSA) (Sigma Aldrich, A8806). Cells were then exposed to the QBT fatty acid uptake dye and changes in fatty acid kinetics were evaluated for 1h using the SprectraMax iD5 Multimode Microplate Reader (Molecular Devices) (excitation 485nm, emission 525nm, Integration 400, temperature: 37°C).

***Masson’s Trichrome staining of aorta and heart cross section:*** Embedded aorta and heart sections stained with Masson’s Trichrome. They were examined under the microscope (Olympus BX40, Olympus America, Melville, NY) at 400X magnification.

***Histological assessment of VAT:*** Unstained VAT was examined under the microscope (Olympus BX40, Olympus America, Melville, NY) at 400X magnification and measured cell diameter in 10 fields/section/slide using image J software. The average of all examined fields/animal and experimental groups was calculated, and the data are presented as mean ± SEM for each group.

***UHPLC-MS metabolomics and lipidomics*:** Snap frozen tissues were ground to powder (GenoGrinder, SPEX, Metuchen, NJ) and extracted in ice cold methanol:acetonitrile:water (5:3:2 v/v) at a 10 mg of tissue/ml solution through vortexing for 30 min at 4°C, followed by centrifugation at 15,000 g for 10 min at 4°C. Twenty μl of supernatants were collected from each extract for metabolomics and lipidomics analyses. Analyses were performed using a Vanquish UHPLC system coupled online to a Q Exactive mass spectrometer (Thermo Fisher, Bremen, Germany). Samples were resolved over a Kinetex C18 column (2.1 x 150 mm, 1.7 μm; Phenomenex, Torrance, CA, USA) at 25°C using a three minute isocratic condition of 5% acetonitrile, 95% water, and 0.1% formic acid flowing at 250 μl/min, or using a 9 min gradient at 400 μl/min from 5-95% B (A: water; B: acetonitrile both phases coupled either with 0.1% formic acid or 10 mM ammonium acetate for positive and negative ion mode, respectively). Metabolite assignments were performed using MAVEN (Princeton, NJ, USA). Data were analyzed using MAVEN (Princeton, NJ, USA) and Compound Discoverer 2.1 (ThermoFisher). Graphs and statistical analyses were prepared with GraphPad Prism 8.0, GENE E, and MetaboAnalyst 4.0.

Further, using metabolomic and lipidomic raw data log2FC and FDR was calculated and significant (>2log2FC; FDR<0.05) results were analyzed by Ingeunity Pathway Analysis (IPA) software, which generates multi-level regulatory networks that helpful to explain the metabolomics expression changes exhibited in a dataset. This tool enables the discovery of novel regulatory mechanisms by expanding upstream analysis to include regulators not directly connected to targets in the dataset.

***Measurement of arterial stiffness by atomic force microscopy:*** Aorta isolated from WT and G6PD^S188F^ rats were incubated in DMEM or DMEM+CCL5 (1mg/mL) at 37℃ for 72 hours. Atomic Force Microscopy (AFM) was employed to quantify the stiffness of isolated rat aorta. For sample preparation, a microsurgery scissor was used to precisely excise a small piece (approximately 2 x 4 mm) from the descending aorta. Each end of the aorta was affixed to a 35-mm culture dish using adhesive and subsequently immersed in 4 ml of PBS. AFM in contact mode was applied to the aorta using an NX12 AFM system (Park Systems) mounted on a Nikon ECLIPSE Ti2 inverted microscope. The aorta was indented with a three-sided pyramidal tip (8 nm in radius) against a silicon nitride cantilever (spring constant = 0.059 N/m; BL-AC40TS-C2, Oxford Instruments). Approximately 10 force-distance curves were acquired from three different locations within each aorta. The force-distance curves corresponding to each indentation were saved as .tiff files and subsequently uploaded to the XEI software (Park Systems) for further analysis. To analyze the stiffness (elastic modulus), the first 600 nm of horizontal tip deflection was fitted with the Hertz model for a four-sided pyramid.

***Measurement of G6PD activity:*** G6PD activity was measured spectrophotometrically with an assay purchased from Cayman Chemicals, MO. The standard assay buffer contained 1 mM MgCl_2_, 50 mM Tris, pH 8.10 (carefully adjusted with concentrated HCl), 0.1 mM NADP^+^ and 0.2 mM glucose-6-phosphate. Five-microliter aliquots (containing 5 μg of protein) of Aorta, liver and VAT homogenate were pipetted into the wells of a 96-well plate (Fisher Scientific) followed by addition of 200 μl of the standard assay buffer. The absorbance at 339 nm was then measured using a plate reader (Synergy HT, Biotek) immediately and for up to 25 min at 50-s intervals. Background absorbance was corrected by subtracting the value of a blank containing no homogenate from all sample readings, and G6PD activity was determined quantitatively using a molar extinction coefficient of 6220 M^-1^cm^-1^.

***Luminex Measurement of Cytokines:*** VAT samples were prepared using the Cell Lysis Buffer ((Invitrogen, Cat#EPX-99999-000) (50 mg tissue/250 µL lysis buffer). Tissues were then homogenized using the Fisher Scientific PowerGen 125 homogenizer on ice. The resulting homogenate was then centrifuged at 13,000 x g for 10 minutes at 4°C. Total protein content for the collected supernatants were measured using the BSA Bradford method. The ProcartaPlex Multiplex Immunoassay ProcartaPlex Rat Cytokine & Chemokine Panel 22plex was purchased from Thermo Fisher (Invitrogen, Cat#EPX220-30122-901). Magnetic beads were added to each well and washed using the Hand-Held Magnetic Plate Washer (Invitrogen, Cat#EPX-55555-000) for 30 seconds. Then, 1X Universal assay buffer and each sample were added in duplicate. The plate was sealed and incubated at 500 rpm on an orbital shaker for 120 minutes at room temperature. Once finished, the plate was washed again before detection antibody was incubated at 500 rpm on an orbital shaker for 45 minutes at room temperature. There was another set of washing, before SAPE solution was added to each well and incubated at 500 rpm on an orbital shaker for 30 minutes at room temperature. Next, the plate was washed and 1x wash buffer was added to each well and incubated for 5 minutes before the plate was ran on the Luminex 200 using the Luminex xPONENT software. Data analysis was completed using the ProcartaPlex Analysis app on Thermo Fisher Connect.

***Quantitative PCR*:** The RNA of aorta was isolated by crushing the tissue with liquid nitrogen and then homogenizing in QIAzol (Catalog #79306), followed by using the Qiagen RNeasy mini kit (Catalog #74104). The total RNA concentration (ng/µL) was determined by Nanodrop 500 measurement, and the resulting RNA concentration was then normalized to 400 ng RNA. This normalized amount of mRNA was then used to prepare cDNA with the Superscript IV VILI mastermix with ezDNAse enzyme (Invitrogen Catalog #11766050). The cDNA was used with the TaqMan universal master mix (Thermofisher Catalog #4304437) for qPCR in duplicate using the Thermofisher QuantStudio3 RT PCR System. All primers used were purchased from TaqMan (Thermofisher). The mRNA expression was normalized to *Tuba1a* and was then analyzed using the 2^-ΔΔCT^ calculation method, as previously described.

Fig.1: Metabolic reprogramming in visceral fat tissue of wild-type (WT) and G6PD^S188F^ rats on high fat diet (HFD) and normal chow (NC) is shown.

Fig. 2: (A) IPA Core Analysis predicts numerous diseases and functions changed in response to G6PD mutation and (B) IPA Network Analysis identifies 3 networks (2 in NC and 1 in HFD) that are related to inflammatory response.

Fig. 3: (A, B) Metabolic reprogramming in liver of high fat diet (HFD) and normal chow groups of wild-type (WT) and G6PD^S188F^ rats. (C) Canonical Pathway Function predicted 15 canonical pathways are significantly and differentially changed in response to G6PD mutation. (D) Upstream Analysis result predicted 29 upstream molecules are significantly and differentially changed in response to G6PD mutation. Thresholds: Absolute z-score ≥ 2 and log10(p-value) ≥ 1.3

Fig. 4: Amino acid and carbohydrate metabolism related functions are predicted to be differentially activated in response to G6PD mutation in liver.

Fig. 5: IPA Network Analysis identifies 2 carbohydrate metabolism related networks in response to G6PD mutation in normal chow and additional 2 carbohydrate metabolism associated networks in HFD.

Fig. 6: Histology of liver showing non-alcoholic fatty liver in wild-type (WT) and G6PD^S188F^ rats on high fat diet (HFD) and normal chow (NC). Scale Bar 100 μm.

Fig. 7: Metabolic reprogramming and upstream regulators of metabolic changes in aorta of wild-type (WT) and G6PD^S188F^ rats fed with high fat diet (HFD) vs. normal chow (NC).

Fig. 8: IPA Network Analysis identifies 2 metabolism-related and 1 inflammation and immunological diseases-related networks associated with HFD.

Fig. 9: Top 25 metabolic pathways differentially up- or down-regulated in VAT, liver, and aorta is shown.

Fig. 10: (A) Masson’s Trichrome staining of aorta isolated from 8-9 months old wild-type (WT) and G6PD^S188F^ rats fed with high fat diet (HFD) or normal chow (NC) is shown. Scale Bar: 10 μm. (B, C) Micrographs showing H&E and Mason’s Trichrome staining of coronary artery from WT and G6PD^S188F^ rats on HFD and NC. Scale Bar: 10 μm in B and 100 μm in C.

Fig. 11: PCR results showing expression of genes related to (A) smooth muscle cell phenotype and (B) factors regulating osteogenic phenotype.
